# Implants with hydrophilic surfaces equalize the osseointegration of implants in normo- and hyperglycaemic rats

**DOI:** 10.1590/0103-6440202204793

**Published:** 2022-12-05

**Authors:** Felipe Eduardo Pinotti, Mauricio Andrés Tinajero Aron, Guilherme José Pimentel Lopes de Oliveira, Elcio Marcantonio, Rosemary Adriana Chiérici Marcantonio

**Affiliations:** 1 Department of Diagnosis and Surgery, São Paulo State University(Unesp), School of Dentistry, Araraquara, Brazil.; 2 Department of Periodontology, San Francisco de Quito University (UFSQ), Quito- Equador.; 3 Department of Periodontology and Implantology, School of Dentistry, Universidade Federal de Uberlândia, Uberlândia, Brazil

**Keywords:** diabetes, osseointegration, implant surface

## Abstract

The purpose of this study was to evaluate the effect of a surface modified by blasting and acid attack and maintained in an isotonic solution compared to a machined surface on osseointegration in normo- and hyperglycaemic animals. Sixty-four animals were allocated into 4 groups with 16 animals each, and they were subdivided into two experimental periods (15 and 45 days), with 8 animals in each group. The groups were divided according to the type of implant that was installed in the animals' tibia and the animals’ systemic condition: CM - Machined implants placed in Healthy animals; CH - Hydrophilic implants placed in Healthy animals, HM - Machined implants placed in animals with hyperglycaemia; HH- Hydrophilic implants installed in animals with hyperglycaemia. The following analyses were performed: biomechanical (removal torque), microtomographic (evaluation of the bone volume around the implants- BV/TV), and histomorphometric (evaluation of bone-implant contact BIC% and of the bone formation area between the threads BBT%). It was found that the implants with hydrophilic surfaces presented higher removal torques and quantities of BV/TV% and higher BIC% and BBT% values in normo- and hyperglycaemic animals. The results of this study indicated that the hydrophilic surface accelerates the osseointegration process (~ 15% BIC/BBT at 15-day period), especially in animals with hyperglycaemia. The hydrophilic surface equaled the osseointegration between normo- and hyperglycaemic animals, reversing the negative potential of hyperglycaemia on the osseointegration process.

## Resumo

O objetivo deste foi o de avaliar o efeito de uma superfície modificada por jateamento e ataque ácido e mantida em solução isotônica em comparação a uma superfície lisa sobre a osseointegração de implantes em animais hiperglicêmicos. No estudo foram utilizados 64 ratos, que foram divididos em 4 grupos com 16 animais em cada, e que foram avaliados em dois períodos experimentais (15 e 45 dias), com 8 animais em cada grupo. Os grupos foram divididos de acordo com o tipo de implante que foi instalado na tíbia dos animais e a condição sistêmica dos mesmos: Grupo CM - Implantes maquinados instalados em animais saudáveis; Grupo CH - Implantes maquinados instalados em animais com hiperglicemia; Grupo HM - Implantes hidrofílicos instalados em animais saudáveis, Grupo HH- Implantes hidrofílicos instalados em animais com hiperglicemia. Foram realizadas análises: biomecânicas (contra torque de remoção dos implantes), histométricas (avaliação do contato do osso com os implantes e da área de formação óssea entre as roscas) e microtomográfica (avaliação da área óssea ao redor dos implantes). Foi verificado que os implantes com superfície hidrofílica apresentaram maiores torques de remoção, quantidade de osso ao redor dos implantes e maiores valores de %BIC e %BBT em animais normo e hiperglicêmicos. Conclui-se que a superfície hidrofílica acelera o processo de osseointegração (~15% de %BIC/%BBT aos 15 dias), especialmente em animais com hiperglicêmicos. A superfície hidrofílica igualou a osseointegração entre animais normo- e hiperglicêmicos, revertendo o potencial negativo da hiperglicemia no processo de osseointegração.

## Introduction

The use of osseointegrated implants is one of the main treatments for edentulism and is currently indicated for several clinical situations, precisely because of its high success rate in rehabilitation treatment ^1^; however, some factors acquired by the host during its lifetime can directly interfere with implant osseointegration (e.g., systemic diseases).

Diabetes mellitus is a systemic disease with serious complications that affects the quality of life of patients. One of these complications is related to oral health, since it has been reported that periodontitis is one of the main complications of diabetes ^2^. Therefore, these patients have a higher risk of tooth loss that can later be treated with the installation of dental implants ^3^. Although it has also been reported that the process of implant osseointegration can be altered in hyperglycaemic states ^4^, studies that evaluated the rate of implant osseointegration in diabetic patients were similar for patients with good glycaemic control compared to patients with inadequate glycaemic control ^5^ ^,^ ^6^.

However, other studies have demonstrated an adverse effect of diabetes on the osseointegration process, especially when the glycaemic control of the host is inadequate. Preclinical studies have shown that hyperglycaemic animals have a delay in osseointegration compared to normoglycaemic animals ^7^, and clinical studies have shown that diabetes mellitus is a risk factor for reducing the survival and success of dental implants ^8^ ^,^ ^9^.

The use of implants with modified surfaces is a good alternative to partially reverse the effect of systemic diseases on bone tissue by accelerating the osseointegration process ^10^. These modified surfaces present some physical and chemical modifications that will positively influence the stability of the blood clot, which is of paramount importance for the osseointegration process, thereby promoting an acceleration in the osteoconduction process ^11^. Among the surface characteristics that interfere with the stability of the blood clot and consequently promote better osteoconduction of the implants, the topography, chemical composition and wettability deserve attention ^12^.

An implant surface modified by blasting and acid etching and then kept in sodium chloride solution has been widely studied and often demonstrates acceleration and improvement of the osseointegration process ^11^ ^,^ ^13^. A preclinical study showed that implants with a hydrophilic surface accelerated the minipig maxillary osseointegration process (49.30 vs. 29.42% at 2 weeks and 81.91 vs. 66.57% at 4 weeks) compared to hydrophobic implants ^13^. A clinical study also showed that implants with hydrophilic surfaces enhance osseointegration compared with hydrophobic surfaces after 28 days of implant placement (48.3% vs. 34.2%) ^14^. In addition, a preclinical study showed that implants with hydrophilic surfaces accelerated the osseointegration process in rat tibiae-grafted areas with different osteoconductive biomaterials ^15^, which are critical areas for the osseointegration process. Thus, the objective of this study was to evaluate the effect of an implant surface modified by blasting and acid etching and immersed in an isotonic solution (hydrophilic) compared to a machined surface on implant osseointegration in animals with induced hyperglycaemia. This study was performed to test the null hypothesis that implants with hydrophilic surfaces can match osseointegration in normo- and hyperglycaemic animals.

## Material and methods

### Ethics considerations, experimental design and groups

The Ethics Committee of Animal Use in Research (FOAr-UNESP - CEUA-44/2017) approved this study. For the study, sixty-four male rats (*Rattus norvegicus*, albinus variation, Holtzman, of approximately 3 months of age, UNESP) were used. The animals were fed solid rat chow and had access to water *ad libitum* before and throughout the experimental period in an environment with controlled humidity, light and temperature cycles.

In this study, the effect of two implant surfaces on osseointegration in normo- and hyperglycaemic animals was evaluated. The 64 animals were randomly divided into 4 groups with 16 animals each, which were evaluated in two experimental periods (15 and 45 days), with 8 animals in each group. The groups were divided according to the type of implant that was installed in the tibia and the systemic condition of the animals: CM - Machined implants (Neodent®, Curitiba, PR, Brazil) placed in Healthy animals; CH - Hydrophilic implants with the surface modified by oxide blasting and acid etching and then kept in sodium chloride solution (Surface ACQUA, Neodent®, Curitiba, PR, Brazil) placed in Healthy animals, HM - Machined implants placed in hyperglycaemic animals; HH- Hydrophilic implants installed in hyperglycaemic animals.

### Hyperglycaemia induction

To induce hyperglycaemia, after a 16-hour fasting period, except for water *ad libitum,* the animals were subjected to intraperitoneal administration of 50 mg/kg streptozotocin dissolved in citrate buffer pH 4.5 thirty days before the surgical procedure. The animals that were kept in a normoglycaemic state received a saline injection intraperitoneally of the same volume. After 24 hours of induction, evidence of hyperglycaemia was obtained by analysing the glycaemic rate. Animals with glycaemia greater than 300 mg/dl were considered hyperglycaemic. Weekly glycaemic level assessments were carried out throughout the study to ensure this condition was maintained.

### Surgical procedure

The animals were anaesthetized with an intramuscular injection of a combination of ketamine (Agener União Ltda, São Paulo, SP, Brazil -dosage of 0.08 ml/100 g of body mass) with xylazine (Rompum, Bayer SA, São Paulo, SP, Brazil - dosage of 0.04 ml/100 g of body weight). After confirmation that the animals were fully anaesthetized, the trichotomy was performed in the region of the animals' tibiae.

An incision of approximately 10 mm was made over the tibial tuberosity, and delicate tissue dissection was performed. Then, the surgical site was prepared for implant placement by means of a progressive sequence of drills (spear drill; 2.0 mm spiral drill - Neodent®; Curitiba, PR, Brazil) to accommodate a 4 x 2.2 mm titanium implant (implants with a machined surface or a hydrophilic surface- Acqua surface, Neodent®, Curitiba, PR, Brazil). The perforations were carried out with the aid of an electric motor, adjusted to 1200 rpm, under abundant irrigation with sterile saline solution. The implants were placed with the aid of a digital key (Hexagonal digital key 1.2 mm - Neodent, Curitiba, PR, Brazil) ^15^.

After the implant placement, the tissues were sutured internally with 5.0 resorbable thread (Vicryl Ethicon, Johnson & Johnson, São José dos Campos, Brazil) and externally with 4.0 silk thread (Ethicon, Johnson & Johnson, São José dos Campos, Brazil). The animals received a single dose of penicillin associated with streptomycin at a dosage of 0.1 ml/kg of body mass (Multibiotic Small, Vitalfarma, São Sebastião do Paraíso, MG, Brazil) and ketoprofen at a dosage of 0.1 ml/kg of body mass (Ketoflex; Mundo Animal, São Paulo, Brazil) intramuscularly.

At 15 and 45 days after the surgical procedure, the animals were sacrificed through an overdose of anaesthetic. The tibiae were randomly separated, one of which was used for microtomographic and histomorphometric analyses, while the other was used for biomechanical evaluation. This selection occurred randomly.

### Biomechanical analysis

After euthanasia, the tibiae selected for biomechanical evaluation were stabilized in a small vice, and a hexagonal wrench was connected to both the implant and the torque wrench (Tohnichi, model ATG24CN-S, Tokyo, Japan - with a graduated scale of 0.05 N/cm, measuring the strength of 3 to 24 Ncm). Anti-clockwise movement was performed to unscrew the implant. The maximum peak required to move the implant was noted as the removal torque value (Ncm).

### µCT analysis

The tibiae selected for this analysis were fixed in 4% paraformaldehyde for 48 hours and subsequently stored in 70% alcohol. To perform the microtomographic evaluation, the tibiae were scanned by a microtomograph (Skyscan, Aatselaar, Belgium) with the following parameters: camera pixel: 12.45; X-ray tube power: 65 kVP; X-ray intensity: 385 µA; integration time: 300 ms; filter: Al-1 mm; and voxel size: 18 µm^3^. After scanning the samples, the images were reconstructed, spatially repositioned and analysed with specific software (NRecon, Data Viewer, CTAnalyser, Aatselaar, Belgium). As the implants placed on the animals' tibias did not have a cover screw, in some cases, bone formation occurred within the prosthetic platform, and since this did not interfere with the bone formation data around the implants, it was necessary to create a region of total interest (ROI) covering the entire region of the implant, which was defined as a circular region 0.5 mm around the entire diameter of the implant. This ROI was defined as the total volume (0.5 mm margin around the implants - 4.5 mm x 3.2 mm), and the second ROI was defined to remove the volume from the implant neck. With the results obtained in the two ROIs, it was possible to define the volume of bone tissue in the ROI using the formula Total Volume - Platform Volume = Volume of bone tissues. The threshold used in the analysis was 25-90 shades of grey, and the values of the volume of mineralized tissue around the implants were obtained as a percentage (BV/TV%). A trained examiner who was blinded to the experimental groups performed this analysis (FEP).

### Histomorphometry analysis

After µCT scanning, the tibiae with the implants were processed by incubation in a graded series of alcohol dehydration (60 - 100%) and subsequently infiltrated and polymerized in light-cured resin (Technovit 7200 VLC, Kultzer Heraus GmbH & CO, Wehrheim, Germany). Using the cut, wear and polish system, the tibia-implant set was processed to obtain non-descaled histological cuts (Exakt Apparatebeau, Hamburg, Germany). The final cuts were approximately 45 μm thick, stained with Stevenel blue associated with acid fuchsin and analysed. The sections were photographed under an optical microscope (DIASTAR - Leica Reichert & Jung products, Wetzlar, Germany) at 100X magnification. Histomorphometric evaluations were performed with ImageJ software (San Rafael, CA, USA). The percentages of bone-implant contact (% BIC) and bone area between threads (% BBT) were assessed separately in the first three threads of the implants. A blinded and trained examiner (FEP) performed these analyses.

### Statistical analysis

All data generated by the analyses in this study were distributed according to the normal distribution as determined by the Shapiro-Wilk normality test. The data were evaluated using the two-way parametric ANOVA test complemented by the Tukey test, which took into account the treatment variables and experimental period. GraphPad Prism 6 software (San Diego, CA, USA) was used for statistical analysis of this study, and all statistical tests were applied at a 5% significance level.

## Results

### Biomechanical analysis (Removal torque)

There was a progressive increase in the removal torque of the implants in all groups over the 45-day period compared to the 15-day period, with the exception of the HH group (p <0.001). Implants with hydrophilic surfaces showed higher values of removal torque than implants with machined surfaces in the control group at the 15-day period (p <0.001) and in the group of hyperglycaemic animals at both evaluation periods (p <0.001). The removal torque was higher in animals in the control group than in animals in the hyperglycaemic group (p <0.001), with the exception of the HH group compared to the CH group at the 15-day time point. The mean and standard deviation of the biomechanical analysis of the implants in all groups and evaluation periods are shown in Table 1.

µCT analysis (BV/TV)

**Table 1:** Mean and standard deviation data from the removal torque analysis (Ncm2) of the implants in all groups and evaluation periods.

Groups x Periods	15 days	45 days
CM	7.71 ± 1.38^B^	21.43 ± 2.69^A*^
CH	17.63 ± 2.50^A^	21.88 ± 2.80^A*^
HM	3.42 ± 2.07^C^	11.75 ± 2.18^B*^
HH	14.29 ± 3.86^A^	17.25 ± 2.49^B^*

Different letters represent differences between groups in each experimental period; * Higher torque than in the 15-day period - Two-way Anova complemented by the Tukey test (p <0.05).

### Microtomographic Analysis

There was a progressive increase in BV/TV values (%) in all groups at the 45-day period compared to the 15-day period (p <0.001). Implants with hydrophilic surfaces showed higher BV/VT values (%) than machined-surface implants in all groups (p <0.001), with the exception of the CM group compared to the CH group at the 45-day time point. The hyperglycaemic animals had lower BV/TV values (%) than the animals in the control group (p <0.001). The mean and standard deviation data from the BV/TV analysis (%) of the implants in all groups and evaluation periods are shown in Table 2.

**Table 2:** Mean and standard deviation data from the BV / TV analysis (%) of the implants in all groups and evaluation periods.

Groups x Periods	15 days	45 days
CM	48.12 ± 1.51^C^	70.21 ± 0.92^A*^
CH	63.57 ± 2.12^A^	70.78 ± 0.98^A*^
HM	31.33 ± 2.05^D^	61.11 ± 1.06^C*^
HH	59.32 ± 2.62^B^	67.31 ± 1.60^B*^

Different letters represent differences between groups in each experimental period; * Higher BV / TV than in the 15-day period - Two-way Anova complemented by the Tukey test (p <0.05)

## Histomorphometry

BIC (%) and BBT (%)

There was a progressive increase in the BIC (%) and BBT (%) in all groups at the 45-day period compared to the 15-day period (p <0.001). Implants with hydrophilic surfaces showed higher BIC (%) and BBT (%) values than machined surface implants in all groups (p <0.001), with the exception of the CM group compared to the CH group at the 45-day period. The hyperglycaemic animals had lower BIC (%) and BBT (%) values than the control group animals with machined implants (p <0.001); however, there were no differences in osseointegration between control and hyperglycaemic animals at the 45-day period when implants with hydrophilic surfaces were placed. The mean and standard deviation of the histometric analysis of BIC (%) and BBT (%) in all groups and evaluation periods are shown in Tables 3 and 4, respectively.

**Table 3:** Mean and standard deviation data from the BIC analysis (%) of the implants in all groups and evaluation periods.

Groups x Periods	15 days	45 days
CM	40.49 ± 5.18^B^	82.60 ± 4.37^A*^
CH	54.26 ± 6.59^A^	85.06 ± 5.52^A*^
HM	21.93 ± 8.37^C^	69.42 ± 5.52^B*^
HH	38.35 ± 5.36^B^	81.01 ± 2.19^A*^

Different letters represent differences between groups in each experimental period; * Higher BIC than in the 15-day period - Two-way Anova complemented by the Tukey test (p <0.05)

**Table 4:** Mean and standard deviation data from the BBT analysis (%) of the implants in all groups and evaluation periods.

Groups x Periods	15 days	45 days
CM	34.79 ± 5.19^B^	81.88 ± 3.10^A^
CH	50.57 ± 5.28^A^	83.22 ± 3.83^A^
HM	11.01 ± 5.10^C^	69.07 ± 4.32^B^
HH	33.88 ± 4.98^B^	79.21 ± 3.39^A^

Different letters represent differences between groups in each experimental period; * Higher BIC than in the 15-day period - Two-way Anova complemented by the Tukey test (p <0.05)

## Discussion

In general, it was verified in this study that implants with hydrophilic surfaces promoted improvement in the osseointegration process compared to implants with machined surfaces and that hyperglycaemia impairs the osseointegration process. However, an interesting finding in this study is that the use of hydrophilic implants equalized the osseointegration process between normo- and hyperglycaemic animals, which, clinically, could mean reducing the importance of glycaemic control for the osseointegration process when using implants with hydrophilic surfaces, which can increase the predictability of treatment of diabetic patients with dental implants.

It was observed in the biomechanical analysis that the secondary stability of the implants was time-dependent and that implants with a hydrophilic surface had higher removal torque values than implants with a machined surface, a finding similar to that observed in other preclinical studies ^15^. In addition, implants installed in normoglycaemic animals showed greater removal torque at 45 days than implants installed in hyperglycaemic animals, even in animals where hydrophilic implants were installed. Although hydrophilic implants accelerate the osseointegration process ^11^ ^,^ ^14^ ^,^ ^15^, it is important to emphasize that the bone remodelling process is continuous and that long periods of hyperglycaemia are associated with deficiencies in bone turnover due to the influence of the action of the terminal products of advanced glycation that impair the function of osteoblasts ^16^, increase the bone tissue reabsorption process ^17^ and impair the formation of bone tissue matrix ^18^.

In contrast, microtomographic and histometric analysis showed that there were no differences in osseointegration and bone formation around the implants between normal and hyperglycaemic animals that were subjected to the installation of hydrophilic surfaces. These similar results in normo- and hyperglycaemic animals specifically associated with the use of implants with a hydrophilic surface may be due to findings from previous studies that demonstrated that these surfaces have a high degree of wettability ^19^, increase the expression of biomarkers for bone formation ^19^, increase the adhesion of undifferentiated mesenchymal cells ^20^, and stimulate differentiation and osteoblastic function ^21^. These properties of the hydrophilic surfaces are related to the improvement in the osseointegration pattern found in preclinical ^11^ ^,^ ^13^ and clinical studies in healthy individuals ^14^ ^,^ ^22^. Furthermore, implants with hydrophilic surfaces accelerate secondary stability in humans ^22^, which can speed up the completion of rehabilitation procedures.

The findings of this study may have valuable applicability in patients with diabetes mellitus, which affects a significant percentage of adults ^23^ and is considered an important risk factor for the loss of implants in function or for osseointegration failures ^24^; however, these effects are dose dependent. In fact, the study by Oates et al., 2009 ^6^ that assessed the stability of implants in healthy patients and type II diabetic patients after 2 and 6 weeks of implant placement showed that the patient's hyperglycaemia was directly correlated with the osseointegration of the implant, and a higher rate of glycated haemoglobin was related to lower stability of the implant. It is important to note that after obtaining osseointegration, even if the implants are installed in compensated diabetic patients, the glycaemic control of these patients may vary during life, and invariably at some point, the implant surface may be challenged by the accumulation of terminal products from advanced glycation ^25^.

This study has some limitations that must be considered when interpreting our data. Rough surfaces have been shown to accelerate osseointegration in general, and although preclinical studies demonstrated that hydrophilic surfaces improve the osseointegration process ^13^ ^,^ ^15^, a clinical study by Khandewall et al., 2014 showed that hydrophilic and hydrophobic implants surfaces implants were no different in achieving osseointegration in diabetic patients, and the real importance of hydrophilic surfaces in this context was limited in our study by the control group receiving an implant with an untreated machined surface ^4^. Another factor to consider is that this study evaluated only the moment of implant installation until the osseointegration phenomenon was obtained. The challenge of this implant to survive under ongoing conditions of hyperglycaemia will continue because implants installed in diabetic patients have shown a greater possibility of being affected by peri-implant disease and have reduced success compared to implants installed in healthy patients ^23^, and this information should be obtained regarding the use of implants with a hydrophilic surface in the future.

## Conclusion

Implants with a hydrophilic surface were observed to accelerate the osseointegration process in normo- and hyperglycaemic animals. This surface was also able to equalize this phenomenon among normo- and hyperglycaemic animals, reversing the negative potential of hyperglycaemia on the osseointegration process.

## References

[1] Papaspyridakos P, Mokti M, Chen CJ, Benic GI, Gallucci GO, Chronopoulos V (2014). Implant and prosthodontic survival rates with implant fixed complete dental prostheses in the edentulous mandible after at least 5 years: a systematic review. Clin Implant Dent Relat Res.

[2] Llambes F, Arias-Herrera S, Caffesse R (2015). Relationship between diabetes and periodontal infection. World J Diabetes.

[3] Conte A, Ghiraldini B, Casarin RC, Casati MZ, Pimentel SP, Cirano FR (2015). Impact of type 2 diabetes on the gene expression of bone-related factors at sites receiving dental implants. Int J Oral Maxillofac Surg.

[4] Khandelwal N, Oates TW, Vargas A, Alexander PP, Schoolfield JD, Alex McMahan C (2013). Conventional SLA and chemically modified SLA implants in patients with poorly controlled type 2 diabetes mellitus--a randomized controlled trial. Clin Oral Implants Res.

[5] Dowell S, Oates TW, Robinson M (2007). Implant success in people with type 2 diabetes mellitus with varying glycemic control: a pilot study. J Am Dent Assoc.

[6] Oates TW, Dowell S, Robinson M, McMahan CA (2009). Glycemic control and implant stabilization in type 2 diabetes mellitus. J Dent Res.

[7] Hua Y, Bi R, Li Z, Li Y (2020). Resveratrol treatment promotes titanium implant osseointegration in diabetes mellitus rats. J Orthop Res.

[8] Al Zahrani S, Al Mutairi AA (2018). Stability and bone loss around submerged and non-submerged implants in diabetic and non-diabetic patients: a 7-year follow-up. Braz Oral Res.

[9] Moraschini V, Barboza ES, Peixoto GA (2016). The impact of diabetes on dental implant failure: a systematic review and meta-analysis. Int J Oral Maxillofac Surg.

[10] Bornstein MM, Schmid B, Belser UC, Lussi A, Buser D (2005). Early loading of non-submerged titanium implants with a sandblasted and acid-etched surface. 5-year results of a prospective study in partially edentulous patients. Clin Oral Implants Res.

[11] Sartoretto SC, Alves AT, Resende RF, Calasans-Maia J, Granjeiro JM, Calasans-Maia MD (2015). Early osseointegration driven by the surface chemistry and wettability of dental implants. J Appl Oral Sci.

[12] Wennerberg A, Jimbo R, Stubinger S, Obrecht M, Dard M, Berner S (2014). Nanostructures and hydrophilicity influence osseointegration: a biomechanical study in the rabbit tibia. Clin Oral Implants Res.

[13] Buser D, Broggini N, Wieland M, Schenk RK, Denzer AJ, Cochran DL (2004). Enhanced bone apposition to a chemically modified SLA titanium surface. J Dent Res.

[14] Lang NP, Salvi GE, Huynh-Ba G, Ivanovski S, Donos N, Bosshardt DD (2011). Early osseointegration to hydrophilic and hydrophobic implant surfaces in humans. Clin Oral Implants Res.

[15] Pinotti FE, de Oliveira G, Aroni MAT, Marcantonio RAC, Marcantonio E, (2018). Analysis of osseointegration of implants with hydrophilic surfaces in grafted areas: A Preclinical study. Clin Oral Implants Res.

[16] Pahwa H, Khan MT, Sharan K (2020). Hyperglycemia impairs osteoblast cell migration and chemotaxis due to a decrease in mitochondrial biogenesis. Mol Cell Biochem.

[17] Graves DT, Ding Z, Yang Y (2000). The impact of diabetes on periodontal diseases. Periodontol.

[18] Hunt HB, Pearl JC, Diaz DR, King KB, Donnelly E (2018). Bone Tissue Collagen Maturity and Mineral Content Increase With Sustained Hyperglycemia in the KK-Ay Murine Model of Type 2 Diabetes. J Bone Miner Res.

[19] Nakazawa M, Yamada M, Wakamura M, Egusa H, Sakurai K (2017). Activation of Osteoblastic Function on Titanium Surface with Titanium-Doped Hydroxyapatite Nanoparticle Coating: An In Vitro Study. Int J Oral Maxillofac Implants.

[20] Zhang Y, Andrukhov O, Berner S, Matejka M, Wieland M, Rausch-Fan X (2010). Osteogenic properties of hydrophilic and hydrophobic titanium surfaces evaluated with osteoblast-like cells (MG63) in coculture with human umbilical vein endothelial cells (HUVEC). Dent Mater.

[21] Gu YX, Du J, Si MS, Mo JJ, Qiao SC, Lai HC (2013). The roles of PI3K/Akt signaling pathway in regulating MC3T3-E1 preosteoblast proliferation and differentiation on SLA and SLActive titanium surfaces. J Biomed Mater Res A.

[22] Velloso G, Moraschini V, Dos Santos Porto Barboza E (2019). Hydrophilic modification of sandblasted and acid-etched implants improves stability during early healing: a human double-blind randomized controlled trial. Int J Oral Maxillofac Surg.

[23] Naujokat H, Kunzendorf B, Wiltfang J (2016). Dental implants and diabetes mellitus-a systematic review. Int J Implant Dent.

[24] Koye DN, Magliano DJ, Nelson RG, Pavkov ME (2018). The Global Epidemiology of Diabetes and Kidney Disease. Adv Chronic Kidney Dis.

[25] Berkowitz RI, Marcus MD, Anderson BJ, Delahanty L, Grover N, Kriska A (2018). Adherence to a lifestyle program for youth with type 2 diabetes and its association with treatment outcome in the TODAY clinical trial. Pediatr Diabetes.

